# *Fab* Advances in *Fabaceae* for Abiotic Stress Resilience: From ‘Omics’ to Artificial Intelligence

**DOI:** 10.3390/ijms221910535

**Published:** 2021-09-29

**Authors:** Dharmendra Singh, Priya Chaudhary, Jyoti Taunk, Chandan Kumar Singh, Deepti Singh, Ram Sewak Singh Tomar, Muraleedhar Aski, Noren Singh Konjengbam, Ranjeet Sharan Raje, Sanjay Singh, Rakesh Singh Sengar, Rajendra Kumar Yadav, Madan Pal

**Affiliations:** 1 Division of Genetics, ICAR-Indian Agricultural Research Institute, New Delhi 110012, India; piyac91@gmail.com (P.C.); chandankrsingh018@gmail.com (C.K.S.); murali2416@gmail.com (M.A.); rsraje@iari.res.in (R.S.R.); 2 Division of Plant Physiology, ICAR-Indian Agricultural Research Institute, New Delhi 110012, India; jyotibiotech86@gmail.com; 3 Department of Botany, Meerut College, Meerut 250001, India; deep.botanydu@gmail.com; 4 College of Horticulture and Forestry, Rani Lakshmi Bai Central Agricultural University, Jhansi 284003, India; rsstomar@rediffmail.com; 5 College of Post Graduate Studies in Agricultural Sciences, Central Agricultural University, Imphal 793103, India; norensingh27@gmail.com; 6 ICAR- National Institute of Plant Biotechnology, LBS Centre, Pusa Campus, New Delhi 110012, India; sanjay_singh777@yahoo.com; 7 College of Biotechnology, Sardar Vallabh Bhai Patel Agricultural University, Meerut 250001, India; sengarbiotech7@gmail.com; 8 Department of Genetics and Plant Breeding, Chandra Shekhar Azad University of Agriculture and Technology, Kanpur 208002, India; rky_csa69@rediffmail.com

**Keywords:** abiotic stress, artificial intelligence, climate change, genetic gain, food legumes, machine learning, omics-assisted breeding, pan-omics

## Abstract

Legumes are a better source of proteins and are richer in diverse micronutrients over the nutritional profile of widely consumed cereals. However, when exposed to a diverse range of abiotic stresses, their overall productivity and quality are hugely impacted. Our limited understanding of genetic determinants and novel variants associated with the abiotic stress response in food legume crops restricts its amelioration. Therefore, it is imperative to understand different molecular approaches in food legume crops that can be utilized in crop improvement programs to minimize the economic loss. ‘Omics’-based molecular breeding provides better opportunities over conventional breeding for diversifying the natural germplasm together with improving yield and quality parameters. Due to molecular advancements, the technique is now equipped with novel ‘omics’ approaches such as ionomics, epigenomics, fluxomics, RNomics, glycomics, glycoproteomics, phosphoproteomics, lipidomics, regulomics, and secretomics. Pan-omics—which utilizes the molecular bases of the stress response to identify genes (genomics), mRNAs (transcriptomics), proteins (proteomics), and biomolecules (metabolomics) associated with stress regulation—has been widely used for abiotic stress amelioration in food legume crops. Integration of pan-omics with novel omics approaches will fast-track legume breeding programs. Moreover, artificial intelligence (AI)-based algorithms can be utilized for simulating crop yield under changing environments, which can help in predicting the genetic gain beforehand. Application of machine learning (ML) in quantitative trait loci (QTL) mining will further help in determining the genetic determinants of abiotic stress tolerance in pulses.

## 1. Introduction

### 1.1. Rationale

Legumes belonging to the *Fabaceae* family are consumed globally and are the second most important food crop after cereals, which are best complemented with the latter to constitute a balanced diet [1]. In some regions of the world, legumes are also utilized as fodder for cattle. Legumes are rich in proteins, vitamins, and minerals and provide bulk to the diet [2,3,4]. Due to their rich nutritional profile, their daily intake can help in reducing micronutrient deficiencies among people in developing countries, who are predominantly impacted by this hidden hunger [5]. Thus, legumes contribute in meeting global food security requirements. Legumes also serve a prospective role in conservative agriculture because of their capability to fix atmospheric nitrogen (N), which improves soil fertility. Early on, most of the legumes were considered to be orphans; however, recent decoding of major food legumes, such as mungbean [6], chickpea [7], common bean [8], soybean [9], pigeonpea [10], cowpea [11], and pea [12], has turned them into rich genomic resources.

Climate change is an unavoidable predicament aggravating abiotic stresses, ultimately threatening global food security by reducing crop yields by around 70% [13,14]. Abiotic stresses, e.g., water stress (e.g., floods and drought), extreme temperature conditions (e.g., heat, cold, and frost), salinity, acidic soils, and heavy metal toxicity, severely affect legume production. To thrive in such harsh conditions, plants counter with strong stress responses. Generally, plants’ stress tolerance is dynamic, involving signal transduction pathways at different regulatory levels to adjust metabolic changes [15,16,17]. These pathways are controlled by several genes, proteins, and post-translational modifications [18,19]. Drought, salinity, and temperature stresses are the major factors that reduce the yield of leguminous crops. These stresses have been aggravated due to the climatic changes over the last few decades [20]. Apart from the most prominent and commonly studied abiotic stresses, there are a few stresses that are more prominent in temperate latitudes that affect phenological abiotic mismatches, which restrict gene flow due to small-scale heterogeneity and affect plant variability. Some phenological mismatches are common in alpine and arctic tundra ecosystems [21]. A restricted gene flow was observed in the long-lived dwarf shrub *Salix herbacea* L. due to variation in the snowmelt timing [22]. Frost stress due to variability in snow cover duration and elevation affected the size and the vulnerability of alpine dwarf shrubs [23,24]. Additionally, variation in altitudinal gradients affected the distribution of *Espeletia* taxa [25] and *S. herbacea* [26]. Flooding in habitats of these mountainous terrains showed variability along with plant traits, such as plant height, plant pubescence, and the presence of aerenchyma, that provided adaptations to variability in alpine environmental conditions [27]. Further, variations in nutrient availability also affect different microhabitats. For example, in the case of *S. herbacea*, there was differential accumulation of nutrients due to plant–soil interactions [28]. This was due to the novel microbial communities that participated in biotic interactions with plants [29]. It is essential to understand the response mechanisms and their regulatory factors to improve pulse production in extremely harsh environments. Since the pathways are regulated at each stage of the central dogma, it is crucial to deploy integrated advanced genomic approaches together with gene editing/transgenic approaches. The former can be best exemplified in the form of ‘pan-omics’, which collaboratively utilizes metabolomic, proteomic, genomic, and transcriptomic data to uncover the precise mechanisms behind stress regulation.

With the emergence of techniques such as next-generation sequencing (NGS) and high-throughput genotyping [30], it is now possible to interpret the precise roles of proteins, genes, and metabolites in legumes. These technologies have also helped in the sequencing and assembly of genomic drafts of major legumes. The details of the genomic drafts of these major legumes are listed in Table 1. High-throughput genomics studies utilizing techniques such as genome-wide association studies (GWAS), genome skimming, genotyping by sequencing (GBS), single nucleotide polymorphism (SNP) chip genotyping, and whole-genome resequencing (WGRS) have been employed in many crops, including legumes, to elucidate the role of stress-responsive genes [31,32]. Further, targeted genome editing is also evolving over time for the development of elite cultivars. Clustered regularly interspaced short palindromic repeats/CRISPR-associated protein 9 (CRISPR/Cas9) as well as other site-directed nucleases, such as transcription activator-like effectors (TALEs) and zinc fingers (ZFs), have emerged as new tools for next-generation breeding [33].

**Figure 1:**
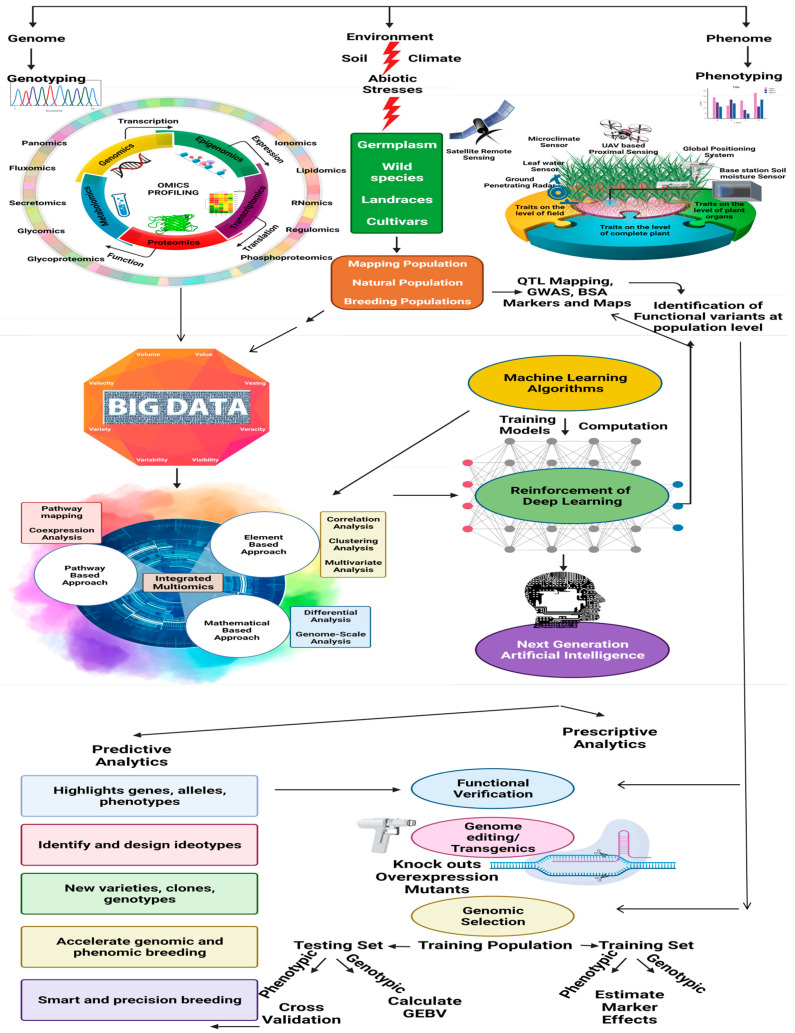
Overall representation of different combinations of omics technologies and artificial intelligence pipelines to predict molecular function-induced abiotic stress. This image was generated by BioRender.com.

Pan-omics and genome editing for the production of climate-smart pulse crops are still new concepts because of the limited availability of genomic information for most of the legumes. Accelerating the development of pulse pan-genomes is therefore, needed for future applications. Based on the advancement of basic omics approaches, the development of some novel omics techniques is gaining momentum. Analysis of metabolic fluxes in the metabolome of an organism is progressing in the form of fluxomics, whereas regulomics is associated with the evaluation of regulatory factors, such as transcription factors (TFs), proteins, and regulatory genes, which are involved in the regulation of gene responses to various abiotic stresses. Likewise, ionomics, glycomics, glycoproteomics, phosphoproteomics, lipidomics, and secretomics represent the advanced omics techniques for studying abiotic stress physiology in different forms [37]. The advent of artificial intelligence (AI) and computer programming for simulations has introduced smart farming as a new facet of climate-resilient crop breeding. Advanced machine learning (ML) algorithms are now being used for crop modeling to obtain maximum yields. Combinations of GWAS and ML algorithms are now being used to detect genetic variants associated with complex abiotic stress tolerance traits [38]. Integration of ML with novel omics techniques will definitely benefit future pulse breeding programs. The overall integration of omics technologies and artificial intelligence pipelines for the molecular functional prediction of abiotic stress tolerance is shown in Figure 1.

### 1.2. Objectives

The present review highlights the opportunities associated with: (i) novel ‘omics’ approaches; (ii) pan-omics approaches; (iii) multi-omics integration; and (iv) AI for smart farming that can handle the climate exigency and its adverse effects on legume production. This will help in the generation of simulation models for future legume breeding and in sustainable agri-production.

## 2. Methods

A systematic review was designed to understand the role of novel omics approaches independently or in association with artificial intelligence in the amelioration of abiotic stresses in legumes and for devising future strategies. The checklist reported in Preferred Reporting Items for Systematic Reviews and Meta-analysis (PRISMA) was followed for an organized assembly of relevant data and information [39]. A comprehensive literature search was performed to identify relevant research articles. More specifically, the papers published until the end of February 2021 in scientific journals were included in this systematic review. Four hundred and twenty seven journals were sorted and added to the list of master journals. We used web search engines such as Google Scholar and Pubmed and, in some cases, websites such as FAO and Knowpulse to search for the information pertaining to legumes’ genomes and production. We searched for the terms “Legumes OR Fabaceae” AND “Omics OR Artificial Intelligence” in titles, abstracts, and/or keywords, which were restricted to articles in the English language, and no date restrictions were imposed. In Google Scholar, articles were sorted by relevance, which included citations, to provide 250 search results. Pubmed yielded 399 results with full-text availability and ‘randomized control trial’ and ‘review’ as the article types. No other relevant article types, such as books and documents, meta-analyses, and systematic reviews, provided any search results. Some studies on plants other than legumes were discarded, although introductory studies on other crops for the development of a particular technology (such as a novel omics technology) were included where necessary. The last search was run on 28 February 2021.

Information on the articles, including the title, abstract, keywords, names of authors, affiliations, journal name, and year of publication, was exported to MS excel. Highly relevant titles and abstracts were then filtered by two independent authors. Thereafter, full-text screenings of these articles for specificity towards the current topic were performed by two reviewers independently. Suggestions, disagreements, and information made by the reviewers to enhance the quality of the present review article were taken into consideration and added to or removed from the main body of the manuscript. The views of both the reviewers were taken into consideration to achieve a consensus. We included all scientific papers that used novel omics approaches or advanced scientific innovations together with any application of artificial intelligence in basic or applied studies on legumes.

## 3. Novel ‘Omics’ Approaches for Future Pulse Breeding Programs

### 3.1. Ionomics

The concept of an ‘ionome’ was first defined by Lahner et al. [40] as the metals, non-metals, and metalloids present in an organism. Later, the term ‘ionome’ was extended to ‘metallome’ [41] to refer to a collection of biologically important non-metals, such as N, phosphorus (P), and sulfur (S). Ionomics is the study of the complete ionome of a tissue/an organism, involving quantification of all elemental constituents in reaction to physiological processes or changes [42]. Ions have a substantial role in the maintenance of a plant’s homeostasis under different environmental conditions. Similarly, ion transporters are important for proper functioning of metabolic pathways as well as in stress regulation. The gene regulatory networks involved in the synthesis of these ions will surely help in furthering our knowledge about the role of ionome in the stress response. An extensive analysis of the *Arabidopsis* genome revealed that around 25,000 genes are engaged in regulating its ionome [40]. Plant ionomics has been extensively reviewed by Baxter (2010) [43], Huang and Salt [44]. A searchable database of more than 22,000 plants mutagenized with fast neutrons or Transfer-Deoxyribo nucleic acid (T-DNA) insertional lines is available at http://hort.agriculture.purdue.edu/Ionomics/database.asp (accessed on 12 January 2021). Similarly, ionome data of 975 soybean lines mutagenized using Nitroso-N-Methylurea (NMU) can be obtained from http://www.ionomicshub.org/home/PiiMS/dataexchange [45]. The *Arabidopsis* ionome project (http://www.ionomicshub.org, accessed on 3 August 2021)with the leaf ionome of more than 125,000 plants is the largest ionomic database till date [43].

The ionomics approach has been extensively used in model legumes such as *Lotus japonicus* [46] and food legumes such as soybean [45,47,48] when compared with other pulse crops. Utilizing this approach, mutants with an altered seed composition were identified in field-grown soybean [30]. Thereafter, they performed GWAS of ionomics traits in the soybean germplasm [47]. A set of 1653 soybean accessions were analyzed for the concentration of 20 elements in the seeds along with their weight. GWAS using oySNP50k chip data and 21 phenotypes showed a multilocus mixed model containing 29 SNPs for iron in one of the three Urbana locations in the year 2009 [47]. Similarly, seed ionome variation in 90 diverse soybean lines was also analyzed [48]. Recent developments in ionomics have provided novel ways to obtain a detailed account of the micro- and macronutrients as well as the elemental composition of legume grains in a rapid and cost-effective manner. The ionome data, thus, can be utilized for studies pertaining to the bioavailability of micronutrients in staple pulses. This way, ionomics can be used to achieve global food security and also to reduce the ‘hidden’ hunger associated with micronutrient deficiencies. The utilization of ionomics for the evaluation of abiotic-stress-responsive ion transporters, genes, ions, and elements requires extensive knowledge of the gene regulatory networks involved in ion homeostasis. Amalgamation of ionomics with other pan-omics approaches, such as proteomics and metabolomics, would increase the opportunities for studying the effects of abiotic stresses in legumes and their applications in producing climate-resilient legumes.

### 3.2. Epigenomics

Epigenomics is gaining importance as an alternate tool for germplasm enhancement. Epigenetic changes that are heritable in nature and affect the cellular processes of an organism form the basis of this tool. This includes modifications such as (de)methylation and (de)acetylation of histones or DNA that do not affect the actual DNA sequence but profoundly affect the gene’s functions [49]. Effects of abiotic stresses on the methylome of many pulse crops have been studied. For e.g., drought stress increased DNA methylation of drought-responsive genes in faba bean and pea [50,51]. Rakei et al. studied the effects of prolonged cold stress on chickpea, which induced DNA demethylation in cold-tolerant genotypes [52]. Similarly, Song et al. reported the consequent effects of salt stress on the epigenome of soybean and found changes in DNA methylation patterns together with histone modifications in salt-stress-responsive transcription factor genes [53]. Liang et al. found that, under continuous cropping stress, DNA demethylation occurred in tolerant soybean genotypes that was consistent with increased expression of demeter-like (DML) and repressor of silencing 1 (ROS1) genes [54]. Wu et al. reported that salinity induced crosstalk between histone methylation and histone acetylation in soybean [55]. In chickpea, salt and drought stresses activated *Ca*HDZ12, a homeodomain leucine zipper (HD-Zip) TF, with acetylation of H3K9ac in the promoter region [56]. Awana et al. found hypermethylation of stress-responsive genes under salinity stress leading to upregulation of salinity-responsive genes in pigeonpea [57]. On the other hand, Chen et al. found hypermethylation of long non-coding ribonucleic acids (lncRNAs) leading to salinity stress tolerance in soybean [58]. Contrary to these studies, increased salinity was found to inactivate some stress-responsive genes in soybean, which was caused by increased deposition of H3K27me3 [59].

Plants gain an epigenetic memory as a result of environmental interactions and pass it on to the next generation. The trans-generational inheritance of epimarks can thus be exploited for crop improvement programs. This involves the use of epialleles, recombinant inbred lines (RILs), and epigenetic quantitative trait loci (epiQTLs) to breed for abiotic stress resistance [60]. Schmitz et al. [61] exploited the epigenetic inheritance of local methyl quantitative trait loci (QTLs) in a soybean RIL population and utilized them to study methyl variations contributing to phenotypic variations over generations. In the same crop, Raju et al. [62] devised an epigenetic breeding strategy utilizing isogenic memory lines crossed to the wild type. The study exploited the amenability of MutS HOMOLOG1 (MSH1), which is responsible for developmental changes such as modulation of defense, the abiotic stress response, and the production of phytohormones, for inducing agronomically important epigenetic variations in soybean. The derived epi-populations of soybean also showed reduced epitype-by-environment (e × E) interactions, representing improved yield stability under changing environmental conditions. Such epigenetic breeding programs can be exploited in other pulse crops for enhancing yield under changing environments.

Comparative epigenomics is an emerging field that provides insights into gene and genome evolution in a similar manner to comparative genomics. Epigenetic mechanisms of gene regulation under abiotic stress may differ between species or may be conserved. Comparative epigenetics is used to understand the evolutionary conservation of the epigenetic regulation of biological functions by comparing epimarks between species [63]. This technique was used to compare the epigenomes of two closely related legumes, namely pigeonpea and soybean. The two genera diverged ~23 million years ago (mya) accompanied by a whole-genome duplication in the latter [64]. The study exploited gene body methylation (GbM) and gene expression patterns to reveal the conservation of nitrogen-metabolism-related genes in the two legumes. Similarly, in another study, methylomes of soybean and common bean were compared to add to the epigenetic resources for leguminous crops [65]. These two legumes share a whole-genome duplication event at around 56.5 mya followed by a genus-specific (*Glycine*) polyploidy event at around 10 mya. Studies on the application of epigenetic breeding in legumes are sparse due to the non-availability of genomic resources. Exploitation of naturally occurring epialleles will fast-track the development of alternate germplasms in orphan legumes with limited genomic information.

### 3.3. Fluxomics

Gathering information on genetic and metabolic regulation through pan-omics has become much easier; however, linking the gathered information to obtain a meaningful crux is difficult. Therefore, combined studies of fluxes through major metabolic pathways controlling the stress response are essential. This necessity has given rise to the study of metabolome-wide fluxes, called fluxomics. This novel omics approach provides the functional output of the cellular machinery involved in stress regulation. Fluxomics can be performed in various ways, including metabolic flux analysis (MFA) and flux balance analysis. The former is concerned with understanding metabolism at the system level under the influence of the environment, whereas the latter is a mathematical model of the metabolism in genomic-scale rebuilding of metabolic networks. MFA can generate metabolic maps that provide details about the metabolic networks involved in the environmental response and represent detailed metabolic phenotypes. Iyer et al. [66] prepared a metabolic flux map from soybean cotyledons to study the consequences of temperature variation for oil and protein biosynthesis using 13 Carbon (C) MFA. The knowledge obtained from metabolic networks can be utilized in the preparation of kinetic models for predicting the effects of environmental factors on genetic changes. Predictive modeling based on fluxomics has been successfully employed in crops such as maize [67] and *Brassica napus* [68]; however, studies on legumes are limited [69]. Moreira et al. developed a metabolic model highlighting metabolic fluxes in soybean seedlings during germination [70]. Similarly, Kannan et al. predicted the cumulative effects of an increase in atmospheric carbon dioxide (CO_2_) on the photosynthesis of soybean using a metabolic model based on gene regulatory networks and metabolic pathways [71]. Fluxomics delineates the key metabolic steps and processes by which fluxes are affected by environmental stresses. Therefore, fluxomics can also be employed to reconstruct metabolic networks in plants for metabolic engineering applications.

### 3.4. RNomics

RNomics is a new omics approach that involves the study of non-coding RNAs, e.g., micro ribonucleic acids (miRNAs) and lncRNAs. MiRNAs are believed to be engaged in stress response regulation in plants. Using NGS, four legume-specific miRNAs (miR5213, miR5232, miR2111, and miR2118) were discovered in chickpea libraries constructed and sequenced for fungal infection, salt treatment, and control conditions [72]. Multiple miRNAs responded under both biotic and abiotic stresses, suggesting the presence of crosstalk between stress-responsive pathways [72]. Barrera-Figueroa et al. [73] reported miRNAs that might have played significant roles in drought tolerance. Likewise, using a homology-based search, Kohli et al. [72] identified various conserved and new miRNAs associated with gene regulation under salt and wilt stress in chickpea.

LncRNAs make up a substantial proportion of non-coding RNAs and are engaged in a variety of biological operations. In one study, PLncPRO, a novel tool, was utilized for predicting lncRNAs in plants using transcriptome data, which revealed a total of 3714 (for drought) and 3457 (for salinity) high-confidence lncRNAs in chickpea [74]. This tool is based on ML and utilizes random forest algorithms to classify coding and long non-coding transcripts. The tool is suitable for plants and has better prediction accuracy compared with existing tools.

### 3.5. Glycomics, Glycoproteomics, and Phosphoproteomics

Glycomics is a comprehensive and developing scientific field that is based on defining the functional and structural roles of glycans in biological systems. Comprehensive knowledge of glycomes is important for understanding biological pathways as glycan modifications are critical to these pathways. The shocking complexity of the glycome, loosely defined as the collection of glycans expressed in a cell/an organism, has resulted in various challenges that must be overcome [75]. Recent advances in mass spectrometry as well as cell and molecular biology tools have helped us address the challenges posed by glycomics. Glycan microarrays are useful in the identification of glycan recognition determinants of glycan protein binding in a system. It is also useful in understanding the functions of glycans and their signaling in a cell or an organism. Moller et al. [76] profiled cell wall glycans in *Arabidopsis* by utilizing a novel technique based on microarrays called comprehensive microarray polymer profiling (CoMPP).

Accurate and high-resolution glycomes can allow for the assignment of an individual glycan molecule that is expressed on a particular glycoprotein. The study of such glycoproteins is called glycoproteomics [77]. The larger the number of glycosylation sites on a protein, the more complex and time consuming the analysis is. In addition, it will require a large amount of sampling material. Advanced techniques for the fragmentation and identification of glycans, such as electron capture dissociation (ECD), ion-trap mass spectrometry (MS), and collision-induced dissociation (CID), have increased the accuracy and feasibility of allocating glycans to specific amino acid sites in a collection of glyopeptides [78]. However, techniques for allocating glycans to specific amino acid sites remain understudied in plants.

Glycoproteomics can unveil the role of protein glycosylation in pulses under stress conditions. In the case of soybean, it was revealed that flood stress negatively impacted the N-glycosylation of functional proteins involved in stress regulation. In contrast, glycoproteins involved in glycolysis were found to be activated [79]. Protein phosphorylation is a key signaling mechanism in the plant abiotic stress response. Phosphoproteomics and glycoproteomics were exploited to study changes under stress conditions in chickpea and soybean [80,81]. Apart from novel molecular techniques, bioinformatics tools focused on glycomics are gaining importance as a new scientific discipline called glyco-bioinformatics. Glyco-bioinformatics utilizes algorithms to study and identify glycans together with their regulation and functions in a system. Recently, Showalter et al. developed a program called BIO OHIO 2.0 to detect hydroxyproline-rich glycoproteins (HRGPs) in the poplar cell wall as well as repeating amino acid sequences, signal peptide sequences, HRGPs, and glycosylphosphatidylinositol lipid anchor addition sequences in other plant species [81]. Similar tools can also be utilized to develop screening platforms for pulses under different stress conditions. Therefore, it is imperative to develop techniques to study protein modifications by glycans in plant cells in order to develop alternate strategies for breeding programs for the enhancement of stress tolerance.

### 3.6. Lipidomics, Regulomics, and Secretomics

Apart from proteins, lipids also play a significant role in stress regulation by maintaining cell wall dynamics under changing environmental conditions. The lipidome, which comprises the lipids expressed in a system, is studied as a subcategory of the metabolome, but its immense importance to cell regulation has made it an emerging scientific discipline [82]. On the other hand, a regulome can be defined as the whole set of the regulatory components present in an organism, including transcription factors, proteins, and mRNAs, which are known to be involved in stress response generation in plants. A few searchable databases are available for analyzing plant regulomes, including Plant Regulomics Portal (PRP) [83] and Plant Regulomics [84], which provide detailed information on transcription factors, small ribonucleic acids (sRNAs), DNA methylation, regulatory elements, gene networks, etc.

Similarly, a plant’s secretome is composed of a group of proteins released into the extracellular matrix that represents the plant’s interaction with its environment [85]. The plant secretome can reveal significant information regarding stress regulation, protein–protein interactions, and defense response generation in a changing environment. Apart from proteins released into the extracellular matrix, protein modifications under abiotic stress also reveal the cellular machinery and cell-to-cell communication in a changing environment. Some of the novel omics technologies described above have been utilized for the enhancement of tabiotic stress tolerance in some legume crops as presented in Table 2.

## 4. Pan-Omics Approaches

Modern biotechnological tools, such as mutagenic breeding, marker-assisted breeding, and transgenic breeding, help in combating the bottlenecks of conventional plant breeding strategies, such as the non-availability of natural resistance and sexual incompatibility in some crops. These can also be utilized to understand the molecular mechanisms of the adaptive response towards abiotic stress(es) in legumes. Genome sequence information is invaluable to the application of next-generation breeding tools in any organism, but it cannot answer some queries related to the gene functions, biochemical pathways, and gene regulatory networks activated during the stress response. Therefore, a more comprehensive approach is required to study the intricate mechanism of the stress response in plants, which should include qualitative and quantitative analyses of gene functions. The knowledge obtained by studying the complex regulatory pathways can be applied in marker-assisted selection (MAS) and transgenic breeding programs for ameliorating the stress tolerance in legumes. Pan-omics integrates the complex omics datasets arising from different omics platforms that can facilitate the improvement of abiotic stress tolerance in crops via precision breeding. The recent progress in pan-omics approaches has remarkably contributed to an enhanced comprehension of the genetic and molecular bases of abiotic stress response generation in many leguminous plants [92].

### 4.1. Genomics

Genomics can be defined as the study of structural, functional, and evolutionary aspects of an organism’s genome. It includes determination of the whole DNA sequence and in-depth genome mapping of an organism. With the advent of NGS and other molecular biology techniques, a large amount of genomics data is available for legumes. Genome sequencing of legume species such as *Lotus japonicus, Glycine max*, and *Medicago truncatula* has already been accomplished [93]. Comparative genomics of these legume crops has revealed key regulatory networks of genes involved in adaptation to stress and crop productivity [94]. Abiotic-stress-related productivity losses in orphan legumes can be managed well using genomic data from the sequenced model legumes. The genomics approach can be linked to marker-assisted backcrossing (MAB) programs for easy manipulation of QTLs associated with stress tolerance and yield parameters. Molecular markers identified using genomics can thus be used in genomics-assisted breeding (GAB) programs, which have higher accuracy than conventional breeding practices [95]. Some of the QTLs identified for various abiotic stresses in legumes are presented in Table 3. Functional genomics techniques, such as insertional mutagenesis, gene overexpression studies, targeted induced local lesions in genomes (TILLING), and gene silencing, play an important role in developing an understanding of the complex gene regulatory networks associated with stress response generation, stress tolerance, and adaptation towards stress in plants. Functional validation of the large amounts of genomics data generated from experiments can be achieved by utilizing reverse genetics and gene silencing approaches such as RNA interference (RNAi), TILLING, and virus-induced gene silencing (VIGS) [96].

### 4.2. Transgenomics

Transgenomics, also known as transgenic technology, is a popular, targeted gene-based technique that provides valuable insights into gene regulation under stress conditions. Foreign genes coding for important agronomic traits from different sources such as plants, animals, and microbes are transferred to the targeted organism’s germline. Many novel phenotypes are developed using transgenomics [126,127]. Transgenic technologies have been employed to elucidate the function of stress-responsive genes in many legumes, such as chickpea [128,129] and soybean [130]. Orphan legumes with limited genetic resources are often utilized in transgenomics for delineating the roles of unknown genes by expressing them in other crops (Table 4).

Several transgenic pulse crops with varying responses to different abiotic stresses have been developed. In transgenic chickpea, miR408 was overexpressed, which resulted in miRNA (miR4080)-induced gene regulation that improved its drought tolerance [150]. The transgenic approach was utilized to develop salinity-tolerant lentils expressing the transgenic *DREB1* gene [151] and mung bean expressing the *Arabidopsis* antiporter (*NHX1*) gene [152]. Additionally, by co-expressing the *Arabidopsis* antiporter (*NHX1*) and *bar* genes in mung bean, Kumar et al. developed salinity-, herbicide-, and oxidative- stress-resistant lines [153]. Many studies have utilized the expression of *Arabidopsis* genes in soybean, such as the *AtMYB44* gene, which resulted in improved drought and salinity tolerance [154] and *AtΔKinase* gene, which resulted in improved salt tolerance [155]. When the mung bean antiporter gene *VrNHX1* was overexpressed in transgenic cowpea, it delivered increased salinity tolerance [156]. Several studies exploited stress-responsive genes from other food crops, such as cereals and vegetables, to improve the overall productivity of legume crops. Kwapata et al. [157] created a drought-tolerant common bean crop using *Hordeum vulgare*’s late embryogenesis abundant (LEA) protein *HVA1*. Likewise, Singh et al. utilized the rice DNA helicase (*OsRuvB*) gene to confer salinity tolerance in pigeonpea [158]. Similarly, Hanafy et al. heterologously expressed the potato gene *PR10a* in faba bean to enhance its salinity and drought tolerance [159]. Transgenic approaches hold a great deal of potential in the development of climate-smart crops, but the lack of proper legislation and the lack of their application in commercial breeding are holding them back from conquering these applications.

### 4.3. Transcriptomics

Transcriptomics is a powerful tool used to quantify gene expression and can provide a precise depiction of the gene expression in a target cell or tissue. Transcriptomics can reveal the gene regulatory networks and candidate genes engaged in abiotic stress response generation, which can be utilized for legume breeding. With the discovery of high-throughput technologies, the deduction of comprehensive transcriptomic data can be executed using serial analysis of gene expression (SAGE) and microarrays. Differential expression of genes (DEGs) can be determined using ribonucleic acid sequencing (RNA-seq) data. A recently developed technique called digital gene expression (DGE) for quantitative estimation of gene expression can also be used. RNA-seq analysis is a cost-effective, high-throughput sequencing technique that makes it possible to analyze large amounts of transcriptomic data. This technique offers several advantages over microarray technology as it does not require genomic information for designing probe sets and can identify novel transcripts [160]. Many studies have exploited this technique for elucidating the gene regulatory networks involved in abiotic stress tolerance in pulse crops (Table 5). Utilizing the NGS approach, a transcriptome atlas has been developed for soybean under drought-stressed conditions [161]. Comparative transcriptomic analysis has described the transcriptional changes in both drought-tolerant and drought-sensitive varieties of soybean [162,163]. Diverse sets of common bean genotypes that were resistant to biotic and abiotic stresses, such as aluminum toxicity, heat, drought, and low phosphorous, were assessed for parental polymorphisms, genetic diversity, and genetic and genomic association mapping using single nucleotide polymorphisms (SNPs) as a marker system, which were derived from Sanger sequencing and Illumina’s GoldenGate technology [164,165,166,167]. Das et al. used metabolomic profiling to reveal that sugar metabolism, nitrogen metabolism, and phytochemical metabolism are of prime significance under water deficit conditions in soybean [168]. From a transcriptomic analysis, Singh et al. identified putative candidate genes expressed under drought stress at the seedling stage in lentil [169], whereas dehydration-responsive proteins were identified by Pandey et al. in chickpea [170]. Molina et al. investigated transcriptomes of chickpea under drought stress using SuperSAGE and deep SuperSAGE and identified 80,238 tags representing 17,493 unique transcripts [171]. Root transcriptome analysis of oxylipin synthesis genes in chickpea unveiled the expeditious induction of jasmonate in roots under drought conditions [172]. Application of RNA-seq for understanding the genes expressed during the stress response will benefit future pulse breeding programs.

The RNA-seq data or microarray data extracted from transcriptome analyses of various crops are used to make high-resolution gene expression atlases (GEAs). GEAs provide information regarding the expression of mRNAs and other important proteins involved in certain biological functions. They act as a valuable resource for studying the expression of genes and proteins engaged in developmental functions as well as in the abiotic stress response. Several GEAs have been developed in pulse crops (Table 6). Apart from GEAs, many transcriptome databases have also been made available for different pulse crops; for example, SoySeq (http://soybase.org/), SoyPLEX (http://www.plexdb.org/plex.php?database=Soybean), and the Chickpea Transcriptome Database (CTDB) (http://www.nipgr.res.in/ctdb.html, accessed on 3 August 2021) [187,188,189]. These extensive transcriptome databases can be used to retrieve data regarding the genes expressed in different tissues in different biological processes under different conditions.

### 4.4. Proteomics and Metabolomics in Abiotic Stress Mitigation

Apart from changes in genes and mRNAs during abiotic stress, plants’ metabolomes and proteomes are also greatly impacted due to these stresses since they are actively involved in defense mechanisms against different stresses [196]. The proteome of an organism, which acts as a bridge between the transcriptome and the metabolome, reflects the actual state of the cellular response better than the DNA markers. The cellular mRNA levels represented by the transcriptome are not accurate depiction of the protein expression as proteins generally undergo post-translational modifications that influence the actual function of proteins [197]. These proteins are of significance to signal transduction pathways and are involved in stress adaptation processes, stress repair mechanisms, etc. Thus, they assist the plant with its recovery from a stress injury and help with its survival under stress [198]. On the other hand, metabolites are a reflection of the gene expression and interactions responsible for gene regulation under stress conditions and have close relations to the phenotype rather than the mRNA or proteins [199]. Of all the different omics technologies, metabolomics is the most cross-functional and reflects most of the processes as they are [196]. Furthermore, metabolic pathways are usually involved in highly complex networks and never function alone, which implies that interrupting a single metabolic pathway, could have adverse effects on other pathways, resulting in damaging traits in the modified plant. Hence, comprehensive analyses that elucidate the metabolic networks involved in the growth and development of plants under varying environmental conditions are very important. The molecular phenotypes of legumes under abiotic stresses have been studied by using proteomics and metabolomics as presented in Table 7.

## 5. Multi-Omics Integration (MOI) for Future Pulse Breeding

**Figure 2:**
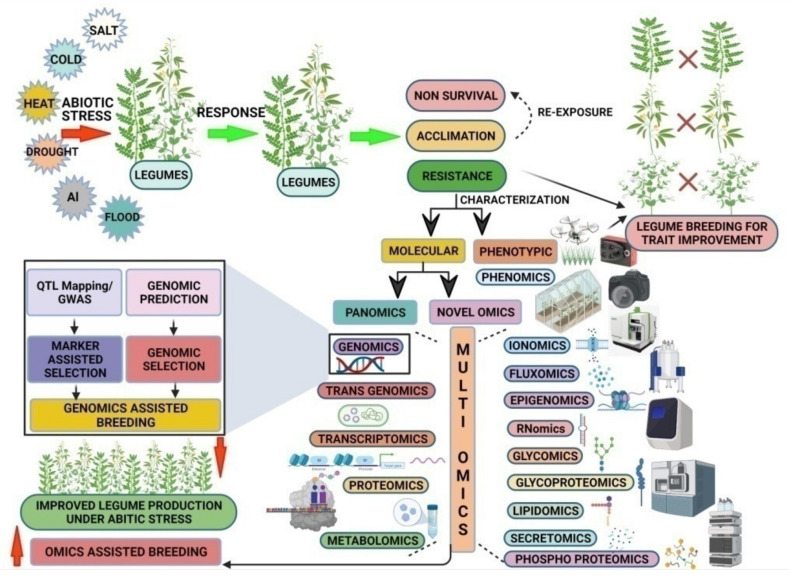
Integrated omics approaches for improving abiotic stress tolerance in legumes. QTL, Quantitative Trait Loci; GWAS, Genome-Wide Association Study. This figure was created by Biorender.com.

Across all disciplines of biology, the rapid development of high-throughput data generation techniques has allowed us to conduct multi-omics-based systems biology research. The data generated from transcriptomics, metabolomics, and proteomics can provide insights regarding the expression of transcripts, metabolites, and proteins, respectively. However, systematic multi-omics integration (MOI) of such data can comprehensively annotate, assimilate, and model these large datasets to provide meaningful, detailed information. Integration of omics data from various platforms together with novel omics approaches can help in bridging the genome-to-phenome gap in crop plants and ultimately help in identifying the right phenotype based on the genetic contribution for breeding purposes [211,212]. The integration of different omics techniques for improving abiotic stress tolerance in legumes is presented in Figure 2.

Large NGS-derived genomic datasets and MOI approaches have substantially contributed towards increasing our knowledge of living organisms at the molecular level. Furthermore, translational genomics (TG) can be used to bridge the information gap between model systems and relatively understudied crop plants. The paramount aim of crop breeding is to achieve the maximum genetic gain of desirable traits in crop genomes in a cost- and time-effective manner. The TG technique has recently been utilized in some of the major legume crops [213].

Recently, GWAS analysis has gained immense popularity due to its ability to find genes, genomic loci, and SNP/InDels in genomes that are associated with beneficial crop traits [214]. Sequencing and/or array-based GWAS tools are making it possible to accurately predict/identify the alleles that are directly linked to particular phenotypic features, which is beyond the reach of map-based QTL analyses. WGRS can reveal genome-wide nucleotide variations, which can be further used for GWAS analyses. Moreover, the development of a high-throughput phenotyping system (HTPS) is imperative for phenotype-associated genomic analyses. Based on their syntenic relationships, the information derived from HTPS can be used for closely related plant genomes. These multi-dimensional and omics-driven techniques can assist with deriving useful information from multi-species phenotypic annotations linked to complex traits. Multi-omics platforms have been integrated together in some legumes to improve abiotic stress tolerance as presented in Table 8.

## 6. Smart Farming: Artificial Intelligence (AI)-Based Pulse Breeding for Climate Resilience

The selection of cultivars with the best traits, especially under stress conditions, requires the modeling of genomics and phenomics data in such a manner that can provide the best output with the minimum cost and effort. MOI data are multidimensional, heterogeneous, and complex data that require advanced solutions for their application in plant breeding technologies. With the advancement of AI technologies, the development of climate-smart crop varieties with enhanced yield can enhance the tolerance/resistance to multiple abiotic stresses and can produce higher genetic gains in less time [222]. A combination of phenotypic, genotypic, and environmental data can reflect a plant’s stress response profile thoroughly; however, due to the complexity of the phenotypic plasticity in changing environments, obtaining meaningful information from integrated data is difficult as it is burdened by the genotype-to-phenotype (GP) gap. Intensive phenotyping involving concurrent comparative phenotypic measurements under changing environmental conditions is required to compensate for unapproachable factors such as the creation of identical growth conditions that are impossible to repeat. The coupling of such measured data with next-generation AI tools will diminish the bias arising from the GP gap. Negin and Moshelion [223] devised a strategy for screening drought-tolerant crops based on the use of a physiology-based high-throughput functional phenotyping system (HFPS) in combination with the soil–plant–atmosphere-continuum (SPAC), which can be used to measure the plant’s response to continuous and fluctuating environmental conditions. The use of a HFPS along with GWAS can result in a better understanding of gene characteristics under changing environments as well as in the development of novel genetic resources for pulse breeding. High-throughput phenotyping in changing environments has also been adopted successfully in certain legumes as presented in Table 9.

### 6.1. Machine learning (ML)-Enabled Genomic Selection, QTL Mining, GWAS, and Functional Prediction for Pulse Breeding

Over the years, GWAS have identified thousands of important genes associated with the stress response. However, due to the complex nature of stress response mechanisms in plants, these responses have been reattributed to multiple interacting genetic variants that are usually ignored in GWAS. ML algorithms can be used to detect these genetic variants. Zhang et al. [38] used a ML-facilitated image phenotyping approach to study the genetic basis of abiotic-stress-related iron deficiency chlorosis (IDC) in soybean. The generated data were subsequently utilized in genomic prediction and GWAS analyses to identify a previously described locus and a new locus containing a gene homolog engaged in iron acquisition. In another study, Naik et al. [230] reported an end-to-end phenotyping approach for soybean stress severity phenotyping that emphasizes IDC-severity-indifferent field plots. The high-throughput framework helped with the digital analysis of stress traits in real-time, identified markers, helped with genomic selection (GS)-based prediction, and increased the rate of genetic gain, which has stress scouting applications in plant breeding as illustrated in the figures of previous works reported by Libbrecht and Noble [231], Schrider and Kern [232], and Cortés et al. [233]. Liu et al. [234] used deep learning technology to predict quantitative phenotypes and to discover markers associated with them. The deep learning framework was based on convolutional neural networks (CNNs), which were used to predict the quantitative traits from SNPs and achieved more accurate results. Similarly, artificial neural networks (ANNs) have been employed for GS-based prediction modeling in common bean [235]. The genotype Aporé, which was studied using ANNs, was recommended for use in unfavorable environments because of its grain yield and high phenotypic stability even under unfavorable conditions. Examples of the use of different machine learning approaches, such as convolutional neural networks, deep belief networks, multivariate Poisson deep learning, multilayer perceptrons, probabilistic neural networks, and radial basis function neural networks, to improve the prediction of tolerance to abiotic stresses, such as heat and drought, in different crop plants have been listed by Cortés and López-Hernández [236]. ML is also a promising tool for QTL mining in crops. Falk et al. [237] developed a computer vision and ML-enabled high-throughput root phenotyping platform for soybean. Using this ML-enabled root phenotyping platform, they studied the genetic variability of root system architecture (RSA) traits in different soybean accessions. The combination of predictive and machine learning algorithms that support genome-wide marker-assisted breeding with innovative methodologies for adaptation to a changing climate together with thermal adaptation has been thoroughly reviewed by Cortés et al. [233,238].

ML systems are cost-effective, non-destructive, and high-throughput tools for the assessment of root growth and development for genomics and phenomics studies. Thus, ML can be efficiently utilized in plant breeding technologies for characterizing the genetic variants controlling complex traits associated with abiotic stress tolerance.

### 6.2. Artificial Intelligence (AI)-Enabled Genome Editing

Genome editing has evolved as an advanced technique to remove deleterious genes from the genome of an organism. Interestingly, the removal of deleterious alleles is one of the important components of plant breeding. Linkage drag can be avoided by the introduction of beneficial alleles into elite cultivars utilizing a genome editing technique rather than backcrossing with other donor parents carrying deleterious alleles at linked loci [239,240]. The utilization of the CRISPR/Cas9 system as a genome-editing tool has opened new avenues in understanding the functional roles of many important regulatory genes. The efficiency of the CRISPR system relies on a specifically designed single-guide RNA (sgRNA) that is complementary to the specific genomic regions under study. However, off-target deletions could result from the binding of sgRNA to off-target sites. AI-enabled identification of target prediction is currently being exploited for designing sgRNAs with increased specificity and improved efficiency. Abadi et al. [241] have designed a computer algorithm using a ML framework called CRISPR Target Assessment (CRISTA) for predicting the target in the genome. The predictions made with CRISTA were found to be more accurate and precise compared with other available methodologies. Most of the existing off-target binding prediction tools are based on the calculation of a mismatch score; thus, they cannot be scaled up with the rapidly increasing amount of experimental data generated through the CRISPR/Cas9 technique [242]. To address this issue, Lin et al. [242] designed two algorithms using deep neural networks, i.e., deep CNNs and deep feed-forward neural networks, to predict off-target mutations in CRISPR/Cas9-based gene editing. The models were evaluated for performance using off-target datasets, such as the CRISPOR dataset (http://crispor.org, accessed on 3 August 2021) and datasets discovered by Genome-wide, Unbiased Identification of DSBs Enabled by Sequencing (GUIDE-seq). The deep neural network-based models were further compared to advanced off-target prediction methods (CCTop, Convolutional Neural Networks, CROP-IT, and MIT) and three conventional ML models (gradient boosting trees, logistic regression, and random forest) in both datasets. The deep neural network-based algorithm made more precise predictions than the conventionally used models. Such ML- and deep-learning-based models can also be utilized in pulse crops for the prediction of off-target binding and, thus, gene editing can also be easily achieved in pulse crops.

## 7. Challenges and Opportunities for Future Pulse Crop Breeding

Legumes share important taxon-specific data opportunities that must be fully explored to improve their abiotic stress resilience. At the individual legume species level, assimilation of novel or unique data is a challenge that can be addressed by integrating different omics approaches and the coupling of phenotyping data with next-generation AI tools. Predictive modeling based on a novel omics approach, such as fluxomics—which can predict the effects of environmental factors on genetic changes—has not yet been explored in case of legumes and should be given attention. In addition, allocating glycans to specific amino acid sites remains understudied in legumes, as tools and algorithms have not yet reached the level of automation required, which needs to be addressed promptly. Furthermore, as large amounts of genomic data on members of the Leguminosae family are becoming available, the creation of comprehensive resource atlases is required. GEAs will be useful for generating markers that are associated with specific productivity- and tolerance-related traits that can be employed in pulse crop breeding. However, many important pulse crops still have a limited number of genetic resources available for the development of databases, which limits the application of epigenetic breeding in legumes. For such pulse crops, the construction of pan-genomes will help us to develop a comprehensive understanding of stress response mechanisms.

Integration of pan-omics platforms with novel omics tools and AI will further assist with the discovery of target genes and pathways controlled by complex mechanisms, which will allow ‘speed cum precision breeding’ to develop climate-resilient, high-yield legumes. However, the MOI approach is often hampered by variations in the data output, data structure, and unwanted noise between the different technological platforms used for data collection. MOI can also be problematic for datasets that are irreproducible, qualitative, contain false positive/negative values, and lack metadata. Therefore, for productive integration and comparison, data management and sharing standards need to be updated. There is a desire to include consistent metadata and ontologies in properly maintained repositories to facilitate their use. Further, genome editing has significantly accelerated livestock breeding; however, genome editing is difficult to achieve in the case of legumes due to the complexity of allelic effects and the GP gap. Computer-simulated, environment-specific models generated from ML- and deep-learning-based models can alleviate the problems associated with genome editing in legumes in changing environments. ML can enable better genomic selection, QTL mining, and genome-wide association studies in orphan legumes. It can also be employed to predict a plant’s response to an abiotic stress by utilizing the miRNA expression in the plant, which to date has only been exemplified in the case of *Arabidopsis* [243]. Similar approaches can also be employed in economically important pulse crops to uncover the role of various stress-responsive miRNAs.

The adjustment of legumes towards changes in climatic scenario and molecular breeding of legumes for resilience to abiotic stresses have conventionally been aided by QTL mapping, marker-assisted selection, and GWAS [244]. Recently, extensive augmentation in the area of predictive breeding has helped us accelerate the selection from natural origins and within the breeding succession by abbreviating the generation intervals and escalating the selection fidelity ahead of field trials. Therefore, predictive breeding has enormous potential for complex polygenic adaptive traits such as abiotic stress tolerance. Lenz et al. have hitherto recognized and talked about refinement in this area, such as multi-trait GP models together with integrative selection scores [245]. These models can describe multi-scale trait–environment inter-relations in legumes. Machine learning provides a predictive method competent at amalgamating GWAS, GEA, and GP approaches. For genetic and genomic datasets, ML algorithms can be dissected into supervised, semi-supervised, and unsupervised methods. Abiotic stress tolerance amelioration requires various ML methods based on the aim of expounding the output model or elucidating the predictive power. Generative models are great for interpretability, whereas discriminative models are suitable for predictive power [231].

Genomic selection also depends upon progress in ML and the accessibility of genotypic data to predict stress-related phenotypic traits. Further scrutiny of the association between mechanistic models that permit the simulation of phenotypes under abiotic stresses and ML models that can incorporate marker data holds potential to solve the problem of model transferability among environments [246]. ML has traditionally been employed in functional genomics [247]. Currently, it is metamorphosing into GWAS coupled to MAS [247] and GP [248,249]. Creative advancements in ML will further assist with precise predictions by agglutinating environmental variables and phenotypic and genotypic diversity [238]. In conclusion, leveraging tools from various scientific disciplines together with “omics” and advanced breeding technologies is crucial to sustaining legume productivity under changing climatic conditions.

## Figures and Tables

**Table 1:** List of published reference genomes of major legumes.

S. No.	Species	Strategy	Accession/Variety/Cultivar	Genome Size (Gbp) Coverage/Estimated	Reference
1.	Chickpea (*Cicer arietinum* L.)	Illumina sequencing of 11 genomic libraries (180 bp to 20 kb)	CDC Frontier, a kabuli chickpea	544.73 (738.09)	[7]
2.	Pigeonpea (*Cajanus cajan*)	Illumina GA and HiSeq 2000 Sequencing system, Sanger-based bacterial artificial chromosome end sequencing	Pigeonpea genotype ICPL 87119 (Asha)	605.78 (833.07)	[10]
2.	Cowpea (*Vigna unguiculata*)	PacBio (Pacific Biosciences of California, Menlo Park, CA, USA) and Single-molecule real-time (SMRT) sequencing	Cowpea IT97K-499-35	519 (613)	[11]
3.	Lentil (*Lens culinaris*)	Illumina sequencing	CDC cultivar Redberry	2600 (4200)	[34]
4.	Mungbean (*Vigna radiata*)	Illumina Hiseq2000 and GS FLX+, with five libraries of a 180-bp fragment, 5, 10, and 40-kb mate-pairs, and one single linear library	VC1973A	543 (579)	[6]
4.	Lotus (*Lotus japonicus*)	Clone-by-clone sequencing and shotgun sequencing	Miyakojima MG-20	315 (472)	[35]
5.	Peanut (*Arachis hypogaea*)	Single-molecule real-time cells (204) run on PacBio RS II system, 14 cells run on the Sequel system, with P6/C4 chemistry	Peanut var. Shitouqi	2540 (2890)	[36]
7.	Soybean (*Glycine max*)	Whole-genome shotgun approach using Sanger sequencing protocols on ABI 3730XL capillary sequencing machines	Soybean var. Williams 82	950 (1115)	[9]

**Table 2:** New omics technologies for pulse breeding.

Pulse	Abiotic Stress	Omics Technology	Details	Reference
Chickpea	Drought	Phosphoproteomics	Phosphorylation of proteins triggered by progressive water deficit conditions.	[80]
		Secretomics	Comprehensive analyses of dehydration, stress-responsive secretome, and highly complex metabolic network function in the extracellular matrix.	[86]
	Oxidative	Secretomics	Role of CaFer1 in iron buffering and adaptation to oxidative stress under changing environmental conditions.	[87]
Common bean	Chlorpyrifos	Lipidomics	Decrease in triacylglycerol levels in pods and seeds.	[88]
Soybean	Heat	Lipidomics	Decreased levels of lipids containing 18:3 acyl chains due to reduced expression of fatty acid desaturase.	[89]
	Low phosphorus	Lipidomics	Lipid remodelling under limited phosphorus conditions.	[90]
	Flooding	Phosphoproteomics	Ethylene signaling pathway played an important role in protein phosphorylation in root tips during flood stress.	[91]
		Glycoproteomics	Flooding negatively impacted the N-glycosylation of proteins.	[81]

**Table 3:** QTLs identified for various abiotic stresses in legumes.

Abiotic Stress	Crop	Parental Lines/Mapping/Genetic Population	Population Type	Trait Studied	Associated Marker(s)	QTLs/Linkage Group(s)	Phenotypic Variation Explained (PVE)	Reference
Drought	Chickpea	ILC 588 × ILC 3279	RILs	Harvest Index, early flowering, and early maturity	97 SSRs	QTLs: Q3-1 and Q1-1 on LG-1 and LG-3, respectively	38%	[97]
		ICC 8261 × ICC 283 and ICC 4958 × ICC 1882	RILs	Root traits	322 SSRs	Main effect (M) QTLs and epistatic (E) QTLs on CaLG01, CaLG02, CaLG03, CaLG04, CaLG05, CaLG06, CaLG07, and CaLG08	M-QTLs: 60% E-QTLs: 90%	[7]
	Cowpea	IT93K503-1 × CB46	RILs	Stem greenness (stg) and recovery dry weight (rdw)	306 AFLP markers	QTL Dro-1-10 (10 QTLs)	For drought related QTLs: 4.7–24.2% For maturity: 14.4–28.9%	[98]
	Common Bean	DOR364 × BAT477	RILs	Photosynthate acquisition, accumulation, remobilization, and other drought-stress-related traits	165 markers (AFLP, RAPD, SSRs)	b03, b05, b06, b08, b09, and b10	37%	[99]
		BRB 191 × SEQ 1027	RILs	Drought-stress-related traits	53 SNPs	Pv10	21%	[100]
		ICA Bunsi × SXB405	RILs	Pod-wall weight, whole-seed weight, whole-pod weight, 100-seed weight	721 SNPs	Pv07	17%	[101]
		BAT 881 × G21212	RILs	Yield components, plant vigor, dry matter redistribution, phenological traits, and mineral nutrients	53 AFLP, 2 RAPD, 42 SSRs, and 127 SNPs	Pv01 and Pv08	12.14–17.24% for the differential stress response	[102]
		SXB412 (A), INB827 (B), ALB213 (C), SEN56 (D), SCR2 (E), MIB778 (F), SCR9 (G), and INB841 (H); 8-way (ABCDEFGH) F1	8-way MAGIC population	Yield, 100-seed weight, iron and zinc accumulation, phenology, and pod harvest index	20,615 SNPs and small indels (< 20 bp)	Pv01, Pv03, and Pv08	35.8 and 5.5% for the major QTL governing hotspot Pv01	[103]
	Lentil	ILL 6002 × ILL 5888	RILs	Dry root weight, lateral root number, taproot length, specific root length, average tap root diameter, root surface area, dry shoot weight, shoot length at 12 and 22 days after sowing, growth rate, seedling vigor, chlorophyll content, root–shoot ratio, and wilting score	220 SNPs and 180 AFLPs	QDRWVII: 21.93, QRSAVII: 21.94, QRSratioIX: 2.30, QLRNVII: 21.94, QSL12IV: 103.83, QSL12VI: 170.87, QSL12VII: 19.71, QDSWVII: 22.94, QSL22VII: 21.94, QLRNIII: 98.64, QSRLIV: 61.63, and QSPADVIII: 72.15.	27.6 and 28.9% for the two consecutive seasons	[104]
	Soybean	Minsoy × Noir 1	RILs	Yield	665 markers (RFLP, SSR)	U14-L, U09-C2, and U11-M	U14-L (20–40%), U09-C2 (14%), and U11-M (23–29%)	[105]
		Pana × PI 567690 Magellan × PI 567731	RILs	Slow wilting	4117 SNPs	*Gm05*, *Gm09*, *Gm12*, *Gm19* *Gm06*, and *Gm10*	7.8–10.4% for *Gm05*, *Gm09*, *Gm12*, and *Gm19*; 20–29.6% for *Gm06*, and *Gm10*.	[106]
	Mungbean	Pagasa 7 × TC 1966	RILs	Drought-related traits	6 AFLPs	-	13%	[107]
		VC2917 × ZL	RILs	Plant height, maximum leaf area, above-ground biomass, relative water content, days to flowering, seed yield, and drought tolerance index	313 SSRs	qPH5A and qMLA2A	qPH5A (6.40–20.06%) and qMLA2A (6.97–7.94%)	[108]
	Pea	P665 × cv Messire	RILs	Drought-related traits	6 SSRs and 2 SNPs	A6, AA175, AC74, AD57, AB141, AB64, Psblox2, PsAAP2_SNP4, and DipeptIV_SNP1	20 to 57%	[109]
Heat	Chickpea	ICC 4567 × ICC 15,614	RILs	Number of filled pods/plot, grain yield/plot, total number of seeds/plot, and percentage of pods set	271 SNPs	CaLG05 and CaLG06	50%<	[110]
	Cowpea	CB27 × IT82E-18	RILs	Heat-stress-related traits	48 SNPs	Cht 5	11.5–18.1%	[111]
	Lentil	JL-3 × PDL-2 and E-153 × PDL-1	F2	Seedling survival and pods set	7 SSRs	qHt_ss and qHt_ps	12.1 and 9.23% for seedling survival and pods set, respectively.	[112]
Cold/Frost	Chickpea	ICC 4958 and PI 489,777	RILs	Cold-tolerance-related traits	747 SNPs	CTCa3.1 and CTCa8.1	7.15 to 34.6% for CTCa3.1 and 11.5 to 48.4% for CTCa8.1	[113]
	Faba bean	Biparental population (BPP): Côte d’Or 1 (French landrace), Bean Pure Line 4628, and Gottingen Winter Bean population	RILs	Frost-tolerance-related traits	5 SNPs	LGs (01, 02, 03, 04, 08, and 10)	2.74 to 29.41%	[114]
	Lentil	WA8649090 × Precoz	RILs	Winter survival traits	94 AFLP, 56 RAPD, 106 ISSR	LG4	22.9%	[115]
	Pea	Champagne × Terese	RILs	Frost tolerance and cold acclimation traits	258 SNPs	LG5 and LG6	6.5 to 46.5%	[116]
	Soybean	Sigalia × Merlin	RILs	Pod number and cold-tolerance-specific traits	7711 SNPs	Chr 11	20%	[117]
Salinity	Chickpea	ICCV 2 × JG-62	RILs	Seed yield, number, weight, flowering time, and shoot dry weight	135 SSR	LG3 (QTL for seed number) LG6 (QTLs for seed number and seed weight) LG4 (QTLs for flowering and shoot dry weight)	19% 14.8–49.7% 8.8–37.7%	[118]
		ICCV 2 × JG 11	RILs	Salinity- and yield-related traits	28 SSRs and 28 SNPs	CaLG05 and CaLG07	12–17%	[119]
	Cowpea	Vignaluteola × V. marina subsp. oblonga	F2	Salt-tolerance- and domestication-related traits	150 SSRs	LG1	20–50.7%	[120]
	Pea	Kaspa × Parafield	RILs	Salt tolerance traits	705 SNPs	Ps III and VII	12% (Ps III) and 19% (VII)	[121]
	Soybean	S-100 × Tokyo	F2:5	Salt tolerance traits	32 SSRs and 116 RFLPs	LG N	29–45%	[122]
Aluminum toxicity	Soybean	Zhonghuang 24 × Huaxia 3	RIL	Al-tolerance-related traits	2639 recombination bin markers (AFLP, RFLP, SSRs)	qRRE_04 and qAAC_04	7.09% (qRRE_04) and 8.98% (qAAC_04)	[123]
		KF No.1 × NN1138-2	RILs	Growth-related indicators for Al resistance, viz. relative total plant dry weight (RTDW), relative root dry weight (RRDW), and relative shoot dry weight (RSDW)	11 SSRs	LG B1	Four additive QTLs (29.39%), four epistatic QTLs (18.75%), and a collective unmapped minor QTL (43.07%)	[124]
		Essex × Forrest	RILs	Physiological traits associated with Al tolerance	14 DNA markers	LG F (Chr. 13)	34%	[125]

AFLP, Amplified Fragment Length Polymorphism; Al, Aluminum; Chr.; Chromosome; ISSR, Inter Simple Sequence Repeat; LG, Linkage Group; MAGIC, Multi-Parent Advanced Generation Intercross; Ps, Photosystem; QTL, Quantitative Trait Loci; RAPD, Rapid Amplified Polymorphic DNA; RFLP, Restriction Fragment Length Polymorphism; RILs, Recombinant Inbred Lines; SNP, Single Nucleotide Polymorphism; SSR, Simple Sequence Repeats.

**Table 4:** Genes and transcription factors (TFs) from different pulse crops overexpressed to generate improved traits or abiotic-stress-tolerant transgenic plants.

Pulse Crop	(A)biotic Stress/Trait	Gene/TF	Gene/TF Family	Transgenic Plant	Reference
Chickpea	Drought and salinity	CaCIPK25 gene	CIPK	Tobacco	[131]
		CAP2 TF	APETALA-2	Tobacco	[132]
		CaHDZ12 TF	HD-zip	Tobacco and Chickpea	[56]
	Drought, salinity, and high temperature	CaZF gene	C2H2-zinc finger	Tobacco and Chickpea	[133]
	Drought	CaAFP gene	Defensin	Arabidopsis thaliana	[134]
		CarNAC2 TF	NAC		[135]
Common bean	ROS stress and wounding	PvACCase gene	Transferase enzyme family	Arabidopsis thaliana	[136]
	Salinity	PvChOMT	O-methyltransferases		[137]
Mung bean	Osmotic stress	VrUBC1 gene	Mung Bean E2 Ubiquitin-Conjugating Enzyme	Arabidopsis thaliana	[138]
Pea	Salinity	p68 gene	DEAD-box protein family	Rice	[139]
				Tobacco	[140]
		PDH45 gene	DNA helicase, initiation factor homologue	Rice and Tobacco	[141]
	Cold, heat, salinity, drought, and freezing	ABR17 cDNA	Group 10 family of pathogenesis-related proteins (PR 10)	Arabidopsis thaliana	[142]
Pigeonpea	PEG, NaCl, cold, and heat	Cajanus cajan cyclophilin (CcCYP), Cajanus cajan hybrid proline-rich protein (CcHyPRP), and Cajanus cajan cold and drought regulatory (CcCDR) genes	Cold- and drought-responsive gene; CYP gene family	Arabidopsis thaliana	[143]
	Drought, salinity, and low temperature	C. cajan cold and drought regulatory (CcCDR) gene	Cold- and drought-responsive gene		[144]
	Drought, salinity, and extreme temperatures	C. cajan cyclophilin (CcCYP) gene	CYP gene family		[145]
	Drought, cold, and salt stress	C. cajan cold and drought regulatory (CcCDR) gene	Cold- and drought-responsive gene	Rice	[146]
Soybean	Drought, salinity, and oxidative stress	GmTP55 gene	Antiquitin-like ALDH7 gene family	Arabidopsis thaliana and tobacco	[147]
	Drought and high salinity	GmDREB2 gene	DREB TF family		[148]
	Drought, high salinity, and resistance to Alternaria alternata, tobacco mosaic virus (TMV), and Ralstoniasola nacearum	GmERF3 gene	AP2/ERF TF family	Tobacco	[149]

HAP2/ERF, APETALA2/Ethylene-Responsive Factor; CIPK, CBL-interacting protein kinases; CYP, Cyclophilin; DNA, Deoxyribo nucleic acid; DREB, dehydration-responsive element binding; HD-zip, homeodomain leucine zipper; LTP, Long-term potentiation; NAC, NAM/ATAF1/CUC2; PEG, Polyethylene glycol; ROS, Reactive Oxygen Species; TF, Transcription factor.

**Table 5:** RNA-Seq for transcriptome profiling of pulse crops under abiotic stress(es).

Crop	Abiotic Stress	Tissue	Sequencing Platform	NCBI BioProject/Accession Number	Details	Reference
Chickpea	Drought	Root and Shoot	Illumina HiSeq 2500	PRJNA396819	TFs associated with drought tolerance were identified.	[173]
		Root	Illumina HiSeq 2500	PRJNA335939	TFs (AP2-EREBP, bHLH, bZIP, C3H, MYB, NAC, WRKY, and MADS) associated with drought tolerance were identified.	[132]
		Leaf	Illumina HiSeq 3000	GSE104609	RNA from leaf tissues at the leaf apical meristem stage was quantified and a total of 1562 genes were differentially expressed in the tolerant genotype. Drought-responsive genes were specifically upregulated in the tolerant genotype.	[174]
	Salinity and drought	Root apex	Roche 454 FLX	PRJNA267525	MiRNA-mediated post-transcriptional regulation of genes engaged in lateral root formation and re-patterning of root hair cells and with high affinity for K+ uptake under salinity and water deficiency conditions was dissected using root apex transcriptome profiling.	[175]
Common bean	Drought	Leaf	Illumina GAIIx	SRR1523069	Drought responsive genes differentially expressed during drought stress were identified.	[176]
	Drought	Leaf and root	Illumina platforms (GAII and HiSeq 2000)	SRP077562	Transcriptome data revealed new genes involved in response to drought stress.	[177]
	Salinity	Cotyledon, hypocotyl, and radicle	Illumina HiSeq 2500 PE 150	PRJNA558376	Role of zinc finger proteins (C3H) was elucidated during the sprouting stage under salinity stress.	[178]
		Root	Illumina HiSeq TM 2000	SRP029243	A total of 2678 TFs were identified from transcriptome data, 441 of which were responsible for salinity tolerance.	[179]
Cowpea	Drought	Leaf	Illumina deep sequencing technology	GSE26402	Exclusive drought-responsive miRNAs were found.	[73]
	Drought	Leaf		GSE20273	A SSH database (http://sshdb.bi.up.ac.za/, accessed on 3 August 2021) was developed for drought-responsive genes.	[180]
	Cold (Chilling)	Pods	Illumina HiSeq 2500	-	sRNAomic and transcriptomic analysis revealed many sRNAs and miRNAs involved in response to chilling.	[181]
Faba bean	Drought	Leaf	Illumina HiSeq 4000	SRX3182042, SRX3182043, SRX3182046, SRX3182047	A total of 538 and 642 putative TFs were identified during the vegetative and flowering stages, respectively.	[182]
		Root	Illumina HiSeq 4000	SRX3182040, SRX3182041, SRX3182044, SRX3182045	Novel DEGs that showed a change in expression during drought were identified.	[183]
	Salinity	Cotyledons	Illumina HiSeq 4000	PRJNA591424	A total of 1410 salinity-responsive genes were identified and significant up-regulation of these genes was observed in the salt-tolerant genotype.	[184]
Lentil	Drought	Leaf	Illumina HiSeq 2500	SRR3105360	Genes involved in oxidation reduction processes, TCA cycle, organ senescence, and reduction of stomatal conductance were more severely upregulated in drought-tolerant genotypes than in drought-sensitive ones.	[92]
	Heat	Leaf	Illumina HiSeq 2000	SUB3390924	Cell wall and secondary metabolite pathways were found to be majorly affected.	[185]
Mung bean	Desiccation	Seed	Illumina HiSeq 2500 with PE125	SRP077637	Many TFs (MYB, AP2, and NAC), HSPs, LEA proteins, and genes encoding methyltransferase and histone were differentially expressed.	[186]

AP2-EREBP, APETALA2/Ethylene-Responsive Element Binding Protein; bHLH, Beta Helix Loop Helix; bZIP, Beta Leucine Zipper; DEGs, Differentially expressed genes; HSPs, Heat shock proteins; LEA, Late embryogenesis associated; MADS, MINICHROMOSOME MAINTENANCE FACTOR1, AGAMOUS, DEFICIENS, and SERUM RESPONSE FACTOR; miRNA, MicroRNA; MYB, myeloblastosis; NAC, NAM/ATAF1/CUC2; RNA, Ribonucleic acid; SSH, Suppression subtractive hybridization; sRNA, small RNA; TCA, Tri carboxylic acid cycle; TFs, Transcription factors.

**Table 6:** High-resolution gene expression atlases (GEAs) for different pulse crops.

Crop	GEA	Details	Reference
Chickpea	CaGEA	The GEA was developed using tissues from 27 samples and RNA studies were done at five different stages, namely the germination, seedling, vegetative, reproductive, and senescence stages. Genes differentially expressed in drought QTL hotspots were also identified.	[190]
Common bean	PvGEA (http://plantgrn.noble.org/PvGEA/)	Regulation of nodulation, nitrogen use efficiency, etc.	[191]
Cowpea	VuGEA (http://vugea.noble.org/)	Conserved regulatory mechanism of miRNAs involved in drought stress and seed maturation.	[192]
Pea	Pea gene atlas portal PsCam (http://bios.dijon.inra.fr/FATAL/cgi/pscam.cgi)	The ‘Caméor’ (PsCam) unigene set allows for the identification of rare transcripts. It can be used to deduce the function of nodulation genes and genes responsible for abiotic stress tolerance.	[193]
Pigeonpea	CcGEA	An important resource for finding candidate genes responsible for specific developmental processes.	[194]
Soybean	http://www.soybase.org/soyseq	An important resource for studying seed filling and developmental genes.	[137]
	http://digbio.missouri.edu/soybean_atlas	A database for comparative analyses with two model legume crops, i.e., *Medicago truncatula* and *Lotus japonicus*, together with the model plant *Arabidopsis*.	[107]
	Small RNA atlas	This atlas helps in identifying novel miRNAs and their targets in the genome.	[195]

**Table 7:** Application of proteomics and metabolomics in abiotic stress mitigation in pulse crops.

Abiotic Stress	Crop	Omics Approach	Details	Reference
Drought	Chickpea	Proteomics	Potential resources for improving drought tolerance were identified.	[200]
		Comparative proteomics	A total of 75 proteins were found to be differentially expressed in roots.	[201]
		Comparative proteomics	MALDI-TOF/TOF-MS/MS analyses revealed 24 differently expressed proteins in leaves under drought stress.	[202]
		Metabolomics	Effect of PGPRs under drought stress was identified using UPLC-HRMS analysis	[203]
	Cowpea	Metabolomics	GC-TOF-MS profiling of primary metabolites and LC-DAD profiling of secondary metabolites under drought stress. Prolonged stress irrespective of the developmental stage affected the metabolome.	[204]
	Faba bean	Proteomics	Proteins including chitinase, Bet, and glutamate–glyoxylate aminotransferase were found to be upregulated in leaves under drought stress.	[205]
Drought and Heat	Soybean	Metabolomics	Upregulation of nitrogen and metabolism under combined heat and drought stress.	[168]
Salinity	Chickpea	Comparative proteomics	Various proteins were found to be engaged in salinity tolerance.	[206]
	Faba bean	Metabolomics	Molecules such as myo-inositol, allantoin, and glycerophosphoglycerol were found to be up-regulated in roots in response to salt stress.	[207]
	Soybean	Comparative metabolomics	A total of 47 different metabolites were found to be responsible for salt tolerance.	[208]
Heat	Chickpea	Comparative proteomics	A total of 482 heat-responsive proteins were found to be engaged in heat stress tolerance.	[209]
Aluminium	Soybean	Comparative proteomics	MALDI TOF analysis revealed differential protein expression in roots under Al stress.	[210]

GC, Gas Chromatography; LC-DAD, Liquid Chromatography with PhotoDiode Array Detection; MALDI-TOF, Matrix-Assisted Laser Desorption/Ionization-Time of Flight; MS, Mass Spectrometry; UPLC-HRMS, Ultraperformance Liquid Chromatography–High-Resolution Mass Spectrometry.

**Table 8:** Multi-omics integration for improved abiotic stress tolerance in pulse crops.

Crop	Stress/Trait/Genes	Pan-Omics Approach Used	Details	References
Chickpea	Abiotic stress	Proteomics and phosphoproteomics	Novel clues suggest that the ubiquitin–proteasome pathway regulates nutrient reallocation. An increased abundance of NAPs/NAPPs involved in redox sensing and signaling during seed development was observed.	[215]
Common bean	Osmotic stress	Proteomics and phosphoproteomics	Dehydrin played an important role in osmotic stress.	[216]
Soybean	Drought tolerance genes	Metabolomics, transcriptomics, and analyses of gene promoters	Metabolite coumestrol and stomatal development genes played important roles in drought tolerance.	[217]
	Silicon transporter involved in (a)biotic stress tolerance	Comparative genomics, transcriptomics, and expression profiling	Two putative Si transporter genes, GmNIP2-1 and GmNIP2- 2, were identified.	[218]
	Salinity	Phosphoproteomics and metabolomics	Flavonoids were significantly upregulated after salt treatment.	[219]
	Salinity	Phosphoproteomics and proteomics	A total of 1163 differentially phosphorylated sites were found, of which ten MYB/MYB transcription factor-like proteins were identified, which were found to be involved in flavonol accumulation.	[220]
	Heat stress tolerance	Genome-wide transcriptomics and proteomics	Proteins involved in thermotolerance, chromatin remodelling, and post-transcriptional regulation under heat stress were identified.	[221]

NAPs/NAPPs, Nutrient-Associated Proteins/Nutrient-Associated Phosphoproteins; MYB, Myloblastosis.

**Table 9:** Automated phenotyping platforms for screening pulse crops in changing environments.

Pulse Crop	Basis of Automated Platform	Details	Reference
Chickpea	Photogrammetry techniques	Open-source 3D phenotyping platform for plant architecture.	[224]
Common bean	Digital imaging techniques	Legume shovelomics—a high-throughput phenotyping platform for common bean and cowpea.	[225]
Cowpea			
Pea	Color imaging technology	High-throughput phenotyping platform for early vigor detection of field pea seedlings responsible for water use efficiency and yield in changing environments.	[226]
	RGB digital imaging	Advanced phenotyping platform for phenotyping pea shoots under cold stress.	[227]
Soybean	Sensor-based technology	Automated phenotyping platform for assessment of salinity in soybean growing under greenhouse conditions.	[228]
	Automated imaging combined with GlyPh	Automated phenotyping platform for predicting drought tolerance in soybeans growing in fields.	[229]

RGB, Red Green Blue; 3D, Three-Dimensional

## Data Availability

Not applicable.
